# Detection of changes in literary writing style using N-grams as style markers and supervised machine learning

**DOI:** 10.1371/journal.pone.0267590

**Published:** 2022-07-20

**Authors:** Germán Ríos-Toledo, Juan Pablo Francisco Posadas-Durán, Grigori Sidorov, Noé Alejandro Castro-Sánchez

**Affiliations:** 1 Tecnológico Nacional de México (TecNM), Campus Tuxtla Gutierrez, Chiapas, Mexico; 2 Instituto Politécnico Nacional (IPN), Ciudad de México, Mexico; 3 Tecnológico Nacional de México (TecNM), Centro Nacional de Investigación y Desarrollo Tecnológico (CENIDET), Morelos, Mexico; Yeungnam University, KOREA, REPUBLIC OF

## Abstract

The analysis of an author’s writing style implies the characterization and identification of the style in terms of a set of features commonly called linguistic features. The analysis can be extrinsic, where the style of an author can be compared with other authors, or intrinsic, where the style of an author is identified through different stages of his life. Intrinsic analysis has been used, for example, to detect mental illness and the effects of aging. A key element of the analysis is the style markers used to model the author’s writing patterns. The style markers should handle diachronic changes and be thematic independent. One of the most commonly used style marker in extrinsic style analysis is n-gram. In this paper, we present the evaluation of traditional n-grams (words and characters) and dependency tree syntactic n-grams to solve the task of detecting changes in writing style over time. Our corpus consisted of novels by eleven English-speaking authors. The novels of each author were organized chronologically from the oldest to the most recent work according to the date of publication. Subsequently, two stages were defined: initial and final. In each stage three novels were assigned, novels of the initial stage corresponded to the oldest and those at the final stage to the most recent novels. To analyze changes in the writing style, novels were characterized by using four types of n-grams: characters, words, Part-Of-Speech (POS) tags and syntactic relations n-grams. Experiments were performed with a Logistic Regression classifier. Dimension reduction techniques such as Principal Component Analysis (PCA) and Latent Semantic Analysis (LSA) algorithms were evaluated. The results obtained with the different n-grams indicated that all authors presented significant changes in writing style over time. In addition, representations using n-grams of syntactic relations have achieved competitive results among different authors.

## Introduction

In a computational linguistics approach, writing style refers to the relative frequency of the use of elements known as style markers. Examples of style markers are frequent words or sequences of words, typing errors, punctuation marks, word length, sentence length, among others. The frequency of use of these markers allows the identification of the writing patterns of a person. Style analysis does not focus on the content of a text, but on the ways in which the author uses language features. Thus, it is possible to use a content-independent markers like grammatical categories, functional words or syntactic structures. Various types of style markers have been proposed for writing style analysis [1, 2]. This work focuses on the use of a style marker known as *n-gram* of different types. An n-gram is a continuous (or even non continuous) sequence of tokens or corresponding elements such as characters, words, Part-Of-Speech tags and syntactic relations [3]. Where *n* is the length of the n-gram. In the context of this research, a change in style refers to changes in the frequency of use of style markers over the time.

Changes in writing styles is important for many problems: diagnosis of neurological diseases [4], authorship attribution [5, 6], author profiling [7, 8], author identification [9] and fake news detection [10, 11].

In this study, we consider n-grams formed by four possible types of elements: characters, words, Part-Of-Speech (POS) tags and syntactic relations. Character n-grams identify the frequency of use at the level of the alphabet of a language: letters, capital letters, punctuation marks or digits. Character n-grams were used with a high performance in many computational linguistic tasks as authorship attribution [12], plagiarism detection [13] and fake news detection [14].

Word n-grams are related to the vocabulary in a document. These features encompass not only the frequency of words, but also vocabulary richness, sentence length, word length distribution and lexical errors. These can be applied to all languages, as the first step of tokenization, i.e., splitting of a text into words. Word n-grams were used, for example, in plagiarism detection [15] and fake news detection [14].

Grammar tags or POS tags assign grammar categories to words according to the context, which they appear. In a sentence, a word can be a verb, noun, pronoun, or adjective among other possibilities. This type of style marker was used for authorship attribution [16, 17], plagiarism detection [18, 19] and fake news detection [20].

We also consider using syntactic n-grams, i.e., n-grams of elements of different types obtained by following the paths in syntactic trees. This concept has been described for example in [21] and was introduced in previous works by this author [22]. In this paper, we only consider syntactic n-grams of dependency relationships. The use of syntactic relations are not entirely conscious, so they seem to be a reliable option for style analysis. Sometimes, usage of syntactic information alone showed poor results in comparison to other types of n-grams [23]. However, other studies have indicated that combining syntactic information with other types of information produces better results, as in authorship attribution [24] and authorship verification [25].

To summarize, n-grams are able to capture stylistic information about an author at lexical, morphological and syntactic levels of a language. We applied the methodology using machine learning. Specifically, we used n-grams of various types as features and then applied the classification algorithms. We divided our data into training and test subsets according to the time periods, perform classification and evaluate the results. If we are able to classify correctly, then there are style changes; otherwise there are no style changes. We also sometimes apply a step of dimensionality reduction using Principal Component Analysis (PCA) and Latent Semantic Analysis (LSA) after choosing the features (n-grams), which is an optional step in the traditional machine learning methodology, namely, we transform the original vector space model into a new one [21].

This is not the first attempt to evaluate the usefulness of syntactic information for detecting changes in writing style over time. Previous works reported the use of features such POS tags n-grams and rewriting rules [5, 23, 24]. Syntactic dependency trees provide information about how a sentence is composed and to the best of our knowledge, none of the related works have experimented with this information. The main contributions of this paper are: 1) compile and annotate a corpus of English authors for a specific task, 2) evaluate the use of syntactic dependency tree n-grams as features for a machine learning approach to detect changes in literary and 3) compare the performance of dependency tree syntactic n-grams with traditional (word, char and POS) n-grams. In addition, this study involves characters, words and morphological information for more reliable results. The effectiveness of using dimensionality reduction techniques to increase machine learning performance is also evaluated.

The rest of this paper is organized as follows. First, we present a brief description of related works. Afterward, we describe the proposed method and detail the performed experiments. In the final section, the results are discussed and interpreted.

## Related work

The idea that everyone has a unique writing style characterized by the selection of the elements and rules used to produce a piece of text are generally accepted. The writing style of an author tends to be preserved regardless of the subject or type of text. However, the writing style can undergo modifications over time caused by social, individual (gender, age and educational level) and geographical factors [26].

Previous works [27, 28] have also studied how mental illness affects writing style, particularly focusing on the Alzheimer’s disease, comparing the novels written at different time periods. The works concentrate on prolific novelists of the British literature, Agatha Christie and Iris Murdoch, both with the Alzheimer’s disease and P.D. James as a control case (without Alzheimer’s disease). In the work [27], it is demonstrated that the disease modifies the style and can be noticeable by phenomena such as a loss in vocabulary and the recurring use of fixed phrases. Experiments conducted by [28] indicated that writing style tends to change over time independent of cognitive decline (as Alzheimer disease). The task of detecting changes in the author’s style has been performed in different scenarios in addition to cognitive decline. The work [29] focused on the task of assigning a date tag to a work (stylochronometry) by identifying changes in the author’s style over different periods of time.

A comparison of the general use of language (diachronic) in an author’s style was accomplished in [30]. Diachronic studies have considered the temporal ordering of an author’s works, seeking to reveal temporal changes within his or her style rather than the changes between authors or between different texts by the same author. They conducted experiments to analyze changes in the author’s style (intra-author) and changes in the styles of two contemporary authors. The authors used multiple linear regression models to predict the year when a text was published. Results indicate that it is possible to identify author’s style changes from the diachronic changes in the use of the language.

The writing style analysis commonly relies on linguistic features, known as style markers. The style markers should be sufficiently robust to allow the identification of an author’s style in all of his works. The most commonly used style markers are frequent words, vocabulary richness, frequent words, function and content words, syntactic complexity, passive voice and POS tags [31, 32].

Commonly, style analysis is approached as a classification task. Style markers should be able to assign text to a correct class. Other issues in style analysis are to determine the amount of text required and the selection of the best markers to obtain better results in the classification.

For reliable style analysis, other factors such as the amount of information in the text (usually measured in words) and the number of available examples should be considered.

Changes in writing style have also been addressed in documents written by more than one author. Thus, the task of style change detection aims at detecting positions of author changes within a collaboratively written text [33]. Since 2017, in the Style Change Detection task, part of PAN at CLEF, participants were asked to detect whether a given document has been authored by multiple authors (up to five) [34, 35].

Other research has used a few texts from a large number of authors. The work [36] used Danish essays written by 10095 authors, with an average of 13 texts per author, to detecting global development trends among students. According to the authors, his approach is based on methods from authorship verification and Siamese Neural Network. The network relies only on character level inputs by using convolutional layers, the network extracts character n-grams (4-grams and 8-grams).

The two main conclusions were: writing style changed more when students start writing more words in their essays and, first year and third year students had higher or equal writing style similarity than two students both in third year, indicating that their writing styles diverge and become more individual. Using some features such as sentence length and word commonness, In [37] conducted a study to examine the changes in the style and content from the Journal of Consulting and Clinical Psychology across time. The authors concluded that Abstracts have changed dramatically across the almost 50 years represented by the data.

The work [38] studied psychological change through mobilizing interactions and changes in extremist linguistic style. The authors stated that the linguistic style is the pattern with which people use function words, which is a collection of non-semantic grammatical word categories. The way people use function words reflects their social psychological states and social relationships.

## Proposed method

### Corpus description

To the best of our knowledge, there is no previous corpus for time style change task. In this paper, we propose a compilation of one (S1 File). The composition of the corpus used for the style analysis is shown in Table 1. This corpus was used in a previous work [39]. It was developed using the novels of 11 native English-speaking authors. Six novels were used per author. Novels were obtained from the Gutenberg Project (https://www.gutenberg.org/). Using the publication date, novels were organized chronologically from the oldest to the most recent. The work of each author was divided into *initial* and *final* stages. All the stages contained 3 novels for each author. Hereafter, the names of the authors are identified as abbreviations, for example BT (Booth Tarkington) or CD (Charles Dickens).

**Table 1:** Corpus description.

	Initial stage		Final stage	
Authors	Year	Title	Year	Title
Arthur Conan (AC)	1887	A Study in scarlet	1917	His Last Bow
Arthur Conan (AC)	1890	The Sign of the four	1926	The Land of Mist
Arthur Conan (AC)	1891	The White Company	1927	Sherlock Holmes
Booth Tarkington (BT)	1899	The Gentleman from Indiana	1919	Ramsey Milholland
Booth Tarkington (BT)	1902	The Two Vanrevels	1921	Alice Adams
Booth Tarkington (BT)	1905	The Conquest of Canaan	1922	Gentle Julia
Charles Dickens (CD)	1838	Nicholas Nickleby	1859	A Tale of Two Cities
Charles Dickens (CD)	1838	Oliver Twist	1861	Great Expectations
Charles Dickens (CD)	1841	Barnaby Rudge	1865	Our Mutual Friend
Edgar Rice (ER)	1912	A Princess of Mars	1941	Llana of Gathol
Edgar Rice (ER)	1914	The Gods of Mars	1942	Skeleton Men of Jupiter
Edgar Rice (ER)	1918	A Warlord of Mars	1944	Land of Terror
Frederick Marryat (FM)	1830	The King’s Own	1845	The Mission
Frederick Marryat (FM)	1831	Jacob Faithful	1847	The Children of the New Forest
Frederick Marryat (FM)	1831	Newton Forster	1848	The Little Savage
George MacDonald (GM)	1863	David Elginbrod	1888	The Elect Lady
George MacDonald (GM)	1864	Adela Cathcart	1891	The Flight of the Shadow
George MacDonald (GM)	1865	Alec Forbes of Howglen	1892	The Hope of the Gospel
Mrs. George de Horne Vaizey (GV)	1901	Tom and Some Other Girls: A Public School Story	1914	Lady Cassandra
Mrs. George de Horne Vaizey (GV)	1902	Pixie O’Shaughnessy	1914	A College Girl
Mrs. George de Horne Vaizey (GV)	1902	A Houseful of Girls	1915	The Independence of Claire
Iris Murdoch (IM)	1954	Under the Net	1985	The Good Apprentice
Iris Murdoch (IM)	1956	The Flight from the Enchanter	1987	The Book and the Brotherhood
Iris Murdoch (IM)	1958	The Bell	1995	Jackson’s Dilemma
John Buchan (JB)	1910	Prester John	1932	The Gap in the Curtain
John Buchan (JB)	1915	The Thirty-Nine Steps	1936	The Island of Sheep
John Buchan (JB)	1916	Greenmantle	1941	Sick Heart River
Louis Tracy (LT)	1903	The Wings of the Morning	1912	One Wonderful Night: A Romance of New York
Louis Tracy (LT)	1904	The Revellers	1916	The Day of Wrath
Louis Tracy (LT)	1905	A Mysterious Disappearance	1919	The Strange Case of Mortimer Fenley
Mark Twain (MT)	1869	The Innocents Abroad	1897	Following the Equator: A Journey around the World
Mark Twain (MT)	1872	Roughing It	1905	What is Man?
Mark Twain (MT)	1876	The Adventures of Tom Sawyer	1906	The 30,000 Dollar Bequest

The detection of changes in writing style over time and authorship attribution task share certain similarities. In both tasks a model was obtained to describe the author’s writing style. We can refer to the available corpus for the authorship attribution task, particularly the closed version of the task, to discuss the size of the proposed corpus. In 2012, the PAN/CLEF evaluation laboratory presented three benchmarks consisting of fragments of novels written by English-speaking authors. Table 2 presents the structure of each PAN benchmark [40].

**Table 2:** PAN/CLEF 2012 benchmark description.

Feature	PAN A	PAN B	PAN C
Authors	3	8	14
Train instances per author	2	2	2
Test instances per author	2	2	2
Size in words (thousands)	1.8 to 6	at most 13	40 to 170

Another corpus was presented in [41], which includes a collection of articles belonging to 13 authors and is grouped into five categories. In this corpus, the number of instances varies by author. The minimum number of instances of an author per category was 1 and the maximum was 10.

The size of the proposed corpus is comparable to that of the corpus examples for the attribution task in two ways: the number of authors and the number of instances per author.

### Preprocessing

All novels were converted into lowercase and divided into sentences using the Natural Language Toolkit (NLTK) available from https://www.nltk.org/. One-word and two-word sentences were discarded because 3-gram words require 3 tokens. To increase the number of examples, the novels were divided into four parts, each with the same number of sentences. Table 3 shows the number of sentences in Booth Tarkington’s novels (BT). Labels 1, 2, 3 and 4 indicate the number of parts into which the novel is divided. For example, the complete novel “The Gentleman from Indiana” has 5,326 sentences. When divided into two equal parts, each text contained approximately 2,663 sentences. By dividing into three equal parts, each text has 1,775 sentences. As the novel is divided into equal parts, the number of examples increases but the number of sentences decreases. The same process was applied to the remaining novels.

**Table 3:** Sentences in novels of BT.

Novels	Number of sentences per sample			
	1	2	3	4
The Gentleman from Indiana	5,326	2,663	1,775	1,331
The Two Vanrevels	2,807	1,403	935	701
The Conquest of Canaan	4,601	2,300	1,533	1,150
Ramsey Milholland	2,180	1,090	726	545
Alice Adams	5,589	2,794	1,863	1,397
Gentle Julia	4,307	2,153	1,435	1,076

### Generation of n-grams

Four types of n-grams were obtained: character, word, POS tag and syntactic relationship. POS tags were obtained by applying POS tagging using the NLTK POS tagger. Stanford Parser [42] was used to obtain syntactic information. The value of *n* indicates the number of tokens in an *n*-gram. Commonly, the values of *n* that have been experimented with are {1,2,3,4,5}. Values higher than 3 could cause the data to become rather sparse [30, 43–45]. As the value of *n* increased, the number of features also increased. In contrast, high-order *n*-grams have very low frequencies of occurrence. These two factors produce sparse data sets. These issues occur regardless of the type of *n*-gram. For all types of *n*-grams, we considered *n* = 3 because this value shows the best performance: plagiarism detection [45], authorship attribution [43, 46], text categorization [47] and author identification [48]. Character, words and POS tags 3-grams were generated with the *text2ngram* (available from https://helpmanual.io/man1/text2ngram/) program, 3-grams of syntactic relations were generated with a script developed in Python [6].

### Creation of document-features matrices

Two groups of document-feature matrices were constructed. In the first group, a 3-gram set was created with a frequency threshold ⩾ 3. Table 4 shows the number of 3-grams obtained using this rule.

**Table 4:** Number of 3-grams per author (in thousands).

Authors	char				words				POS				Syntactic			
	1	2	3	4	1	2	3	4	1	2	3	4	1	2	3	4
AC	5	5	5	4	4	2	1	1	4	4	3	3	5	4	3	3
BT	7	6	6	6	3	2	1	1	5	4	3	3	4	3	3	2
CD	8	7	7	7	32	21	17	14	8	7	7	6	8	6	5	5
ER	4	4	4	3	4	2	2	1	3	3	2	2	4	3	3	3
FM	8	7	7	7	9	6	4	3	6	5	4	4	5	4	3	3
GM	8	7	7	6	7	4	3	2	6	5	4	4	6	4	4	3
GV	7	6	6	5	3	1	1	1	4	4	3	3	4	3	3	2
IM	6	6	6	5	10	6	5	4	6	5	5	4	6	5	5	4
JB	5	5	5	4	4	2	2	1	4	3	3	3	4	3	3	3
LT	7	6	6	5	3	1	1	1	4	3	3	3	5	3	3	3
MT	9	8	7	7	7	5	4	3	6	5	4	4	5	4	3	3

In machine learning, if the analyzed objects have a large number of characteristics, it is convenient to reduce their number [49]. This could improve the results of the machine learning metrics (precision, recall, accuracy and F1). The second group of matrices was obtained by applying *dimensionality reduction* techniques to the first group. Dimensionality reduction was performed using PCA and LSA algorithms, both implementations of *scikit-learn* [50].

The dimensionality reduction process can be defined as follows: Given a matrix A of *m* × *n*, where *n* is large; it is often desirable to project the *m* lines to a smaller dimensional space, to a matrix of *m* × *n*, with *k* < *n*, where *k* represents the new dimensions of the matrix. It is difficult to determine the appropriate value of *k*, because it depends on the dataset. A common heuristic for estimating *k* involves setting a threshold. In this analysis, experiments were carried out using two strategies: (1) selecting *k* dimensions where *k* is the number of samples in the training set and (2) selecting the *k* most informative features (commonly *k* = 2).

### Experimental settings

The process of creating the training and test sets is described as follows. As an example, Table 5 shows the novels of author BT that were used in the experiments.

**Table 5:** Novels of author BT.

Initial stage		Final stage	
Novel	Year	Novel	Year
The Gentleman from Indiana	1899	Ramsey Milholland	1919
The Two Vanrevels	1902	Alice Adams	1921
The Conquest of Canaan	1905	Gentle Julia	1922

The data were divided into training and test sets using the Leave-One-Out strategy, i.e., a novel per class was used once as a test set and the remaining novels were used for the training set. Thus, nine training and test tuples were created for each author. Table 6 shows the test and training sets for the author BT.

**Table 6:** Test and training sets of BT.

Novels	Run 1		Run 2		Run 3		Run 4		Run 5		Run 6		Run 7		Run 8		Run 9	
	Train	Test	Train	Test	Train	Test	Train	Test	Train	Test	Train	Test	Train	Test	Train	Test	Train	Test
The Gentleman from Indiana		★		★		★	✔		✔		✔		✔		✔		✔	
The Two Vanrevels	✔		✔		✔			★		★		★	✔		✔		✔	
The Conquest of Canaan	✔		✔		✔		✔		✔		✔			★		★		★
Ramsey Milholland		★	✔		✔			★	✔		✔			★	✔		✔	
Alice Adams	✔			★	✔		✔			★	✔		✔			★	✔	
Gentle Julia	✔		✔			★	✔		✔			★	✔		✔			★

Dividing novels into fragments increased the number of instances. The distributions of the test and training sets are listed in Table 7. A complete novel is always taken regardless of the number of parts it is divided into. A proportion of 1/3 (≈ 33%) was used for testing and 2/3 (≈ 67%) for training.

**Table 7:** Distribution for training and test sets.

Size	Samples	Sets	
		Test	Training
1	6	2	4
2	12	4	8
3	18	6	12
4	24	8	16

The classification tests were performed with well-known Logistic Regression (LR) and Support Vector Machine (SVM) supervised machine learning algorithms, implemented with the *scikit-learn* library. The accuracy metric is defined as the fraction of predictions correctly made by the model. This metric is not a good choice when there is class imbalance [51]. However, in these experiments both classes were balanced, so the accuracy was appropriate for the evaluation [52]. Precision and recall metrics were used to obtain reliable results. Their mathematical representations are presented in Eqs 1, 2 and 3.

$$\begin{array}{c} accuracy=\frac{TP+TN}{TP+TN+FP+FN} \end{array}$$ (1)

$$\begin{array}{c} precision=\frac{TP}{TP+FP} \end{array}$$ (2)

$$\begin{array}{c} recall=\frac{TP}{TP+FN} \end{array}$$ (3)

The problem was addressed as a supervised authorship attribution: given a document D and two stages S = {*Initial*, *Final*} for a unique author, determine to which of the two stages in S, D belongs. This is a binary classification problem where positive class is labeled with the *Initial* tag. The binary classifier predicts instances of the test set as positive or negative and produces four outcomes: True Positive (TP), True Negative (TN), False Negative (FN) and False Positive (FP).

A simple and natural way to view text is as a sequence of items (words, digits and punctuation marks) grouped into sentences. Based on words, a text is a selection of words used by an author to express an idea. This set of words is known as a vocabulary. How often use and the way in which it combines these words provide clues to authorship. Therefore, we propose as a baseline a word 3-grams model.

## Results of experiments

**Fig 1:**
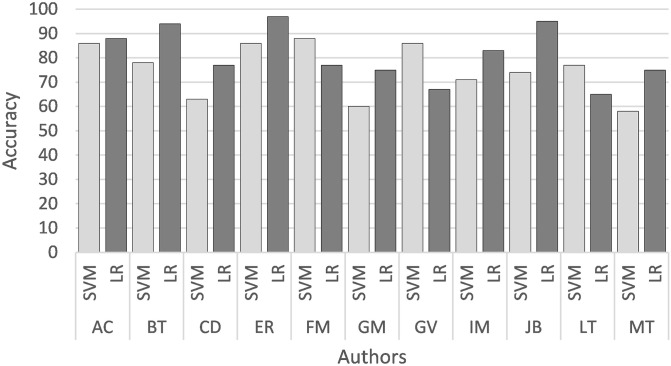
Average accuracy (%) of SVM vs LR.

In the first stage, both learning algorithms were evaluated to determine the general average accuracy using complete novels. Fig 1 shows the average of the four groups of 3-grams and the SVM and LR classifiers. In general, LR outperformed SVM. Therefore, the presentation of results continues with the LR classifier.

The results of the experiments are presented and identified with the label ALL-features (experiments without dimensionality reduction) and PCA-features and LSA-features (experiments with dimensionality reduction techniques). Labels 1, 2, 3, and 4 indicate the number of parts the novel has been divided into.

### All-features results

The results of the experiments for 3-gram using ALL-features are listed in Table 8. Complete novels (1) achieved a higher accuracy than other sample sizes. The accuracy exceeds 70% for most of the authors, except the case of the author GV in character (here and after char), words and POS 3-grams.

**Table 8:** Accuracy (%) using ALL-features with different split settings.

Authors	char				words				POS				Syntactic Relationship			
	1	2	3	4	1	2	3	4	1	2	3	4	1	2	3	4
AC	78	75	74	75	100	75	39	42	67	78	69	66	83	81	80	82
BT	94	92	94	96	100	97	83	81	100	97	96	97	83	83	80	78
CD	61	67	67	70	89	89	83	86	78	92	91	90	89	92	91	86
ER	100	100	100	100	89	86	89	82	100	100	94	96	100	94	92	92
FM	67	72	76	76	78	72	67	66	78	81	82	84	89	92	85	89
GM	72	78	76	77	72	69	61	74	61	47	58	56	89	86	85	85
GV	50	50	46	45	67	67	63	61	61	67	65	74	83	78	80	79
IM	89	83	81	81	100	100	98	96	89	89	89	82	100	100	100	96
JB	89	86	87	85	100	92	89	86	100	83	89	85	100	92	94	92
LT	72	67	65	65	61	64	61	49	61	64	61	49	50	50	56	53
MT	61	58	70	70	83	83	83	78	78	78	74	71	78	83	78	81

**Fig 2:**
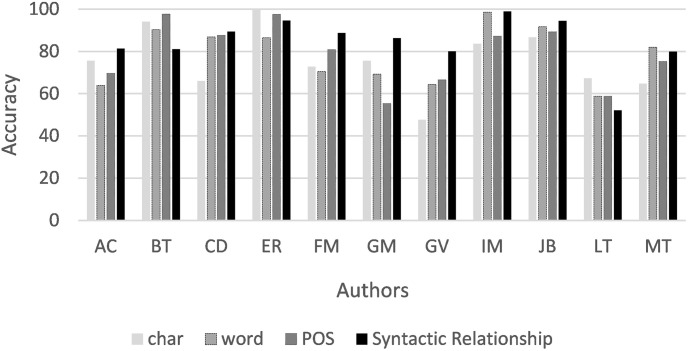
Average accuracy (%) of each type of n-gram for the authors.

The authors ER, IM, and JB achieved 100% accuracy on Syntactic Relationship 3-grams and on one of the remaining 3-grams. In particular, LT exhibits the lowest accuracy in the experiments. However, Fig 2 shows that the average accuracy of syntactic 3-grams was higher in 8 of the 11 authors.

**Fig 3:**
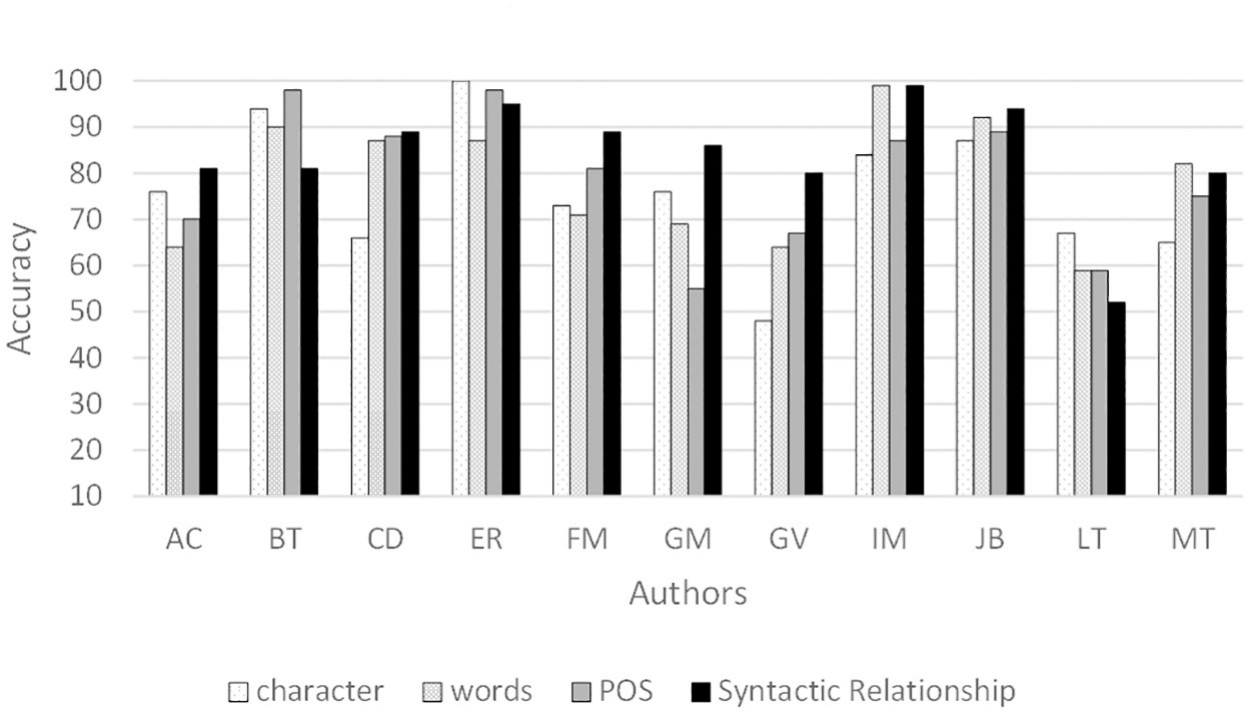
Average accuracy (%) for 3-grams using all features.

Fig 3 shows the average accuracy achieved for the different types of 3-grams. 3-grams of syntactic relations achieved the highest accuracy for most authors.

### PCA-features

Two approaches were tested for selecting *k*. First, using *k* = {4,8,12,16}, these values corresponded to the number of samples in the training set. Second, we used a fixed value (*k* = 2). The latest approach yielded the best results; therefore, it was the reported approach.

The results of the 3-gram using PCA features are listed in Table 9. Complete novels (1) showed slightly better results than the other sample sizes. The authors AC and MT just achieved only the 50% accuracy through the different experiments. Similarly, CD and GM achieved a maximum accuracy of 56% in character 3-grams and complete novel(1). For POS tags and syntactic relationship 3-grams, the authors ER, FM, IM, JB and LT have accuracies ranging from 80% to 100%.

**Table 9:** Accuracy (%) using PCA-features (1, 2, 3, 4 are split settings).

Authors	char				words				POS				Syntactic Relationship			
	1	2	3	4	1	2	3	4	1	2	3	4	1	2	3	4
AC	44	44	46	50	44	44	43	38	50	58	52	54	50	58	56	57
BT	100	89	91	82	89	86	85	70	94	89	85	82	56	56	54	52
CD	56	53	52	54	83	81	68	72	67	67	68	64	56	58	52	53
ER	100	98	98	97	78	72	65	61	100	100	98	99	100	100	100	100
FM	89	92	91	90	78	72	59	67	100	100	96	95	83	86	87	89
GM	56	67	78	76	83	83	83	82	56	75	72	75	67	81	78	68
GV	100	92	92	89	78	78	80	77	100	83	96	78	72	75	76	76
IM	83	78	76	74	83	78	80	79	61	61	61	58	83	83	83	83
JB	72	61	52	46	100	97	94	93	78	78	67	61	89	86	80	77
LT	78	78	76	71	78	58	63	61	67	72	63	57	100	100	98	97
MT	50	44	48	47	50	39	39	49	56	56	54	56	50	42	37	38

**Fig 4:**
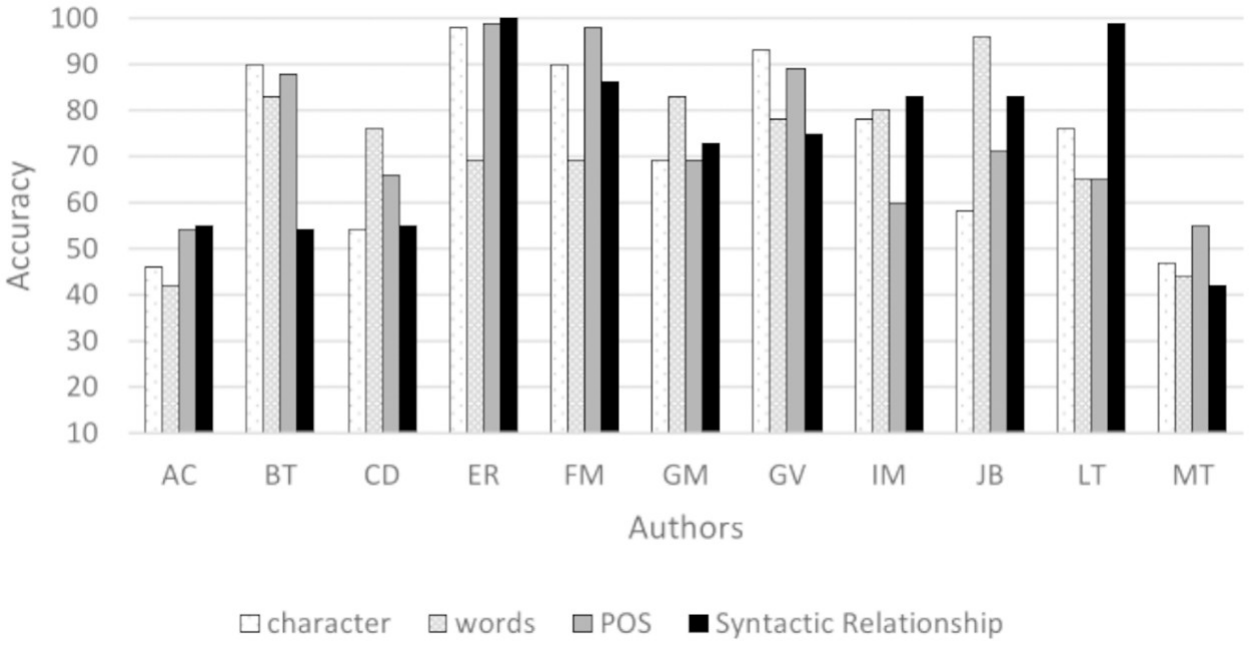
Average accuracy (%) for 3-grams using PCA-features.

Fig 4 shows the average accuracy achieved for the four text sizes. In some cases, syntactic relationship 3-grams are surpassed by any of the other 3-grams, especially for authors CD, FM and IM. For the rest of the authors, all 3-grams obtain similar results.

### LSA-features

Similarly, two proposed approaches for selecting the value of *k* were tested in LSA. First, using *k* = {4, 8, 12, 16}, these values correspond to the number of samples in the training set. Second, using a fixed value (*k* = 2). The second approach yielded the best results; therefore, it was the reported approach.

The results for 3-grams using LSA features are shown in Table 10. Complete novels (1) showed slightly better results than the other text sizes. In character and word 3-grams, the authors BT, ER, IM and JB achieved results greater than 70% accuracy. The authors AC and GV showed the highest accuracy in syntactic relationship 3-grams, even authors such as ER, IM and JB achieved 100% accuracy. On the other hand, LT achieved the highest accuracy in POS 3-grams, with only 72% accuracy. Similar to ALL-features (See Fig 2), the average of the Syntactic Relationship 3-grams slightly exceeds the other 3-grams in 8 of the 11 authors.

**Table 10:** Accuracy using LSA-features (1, 2, 3, 4 are split settings).

Authors	char				words				POS				Syntactic Relationship			
	1	2	3	4	1	2	3	4	1	2	3	4	1	2	3	4
AC	61	69	69	67	56	56	41	43	67	69	76	75	89	83	83	85
BT	72	92	94	96	56	78	81	78	89	92	92	95	67	72	69	74
CD	56	64	67	71	50	64	83	83	72	89	91	92	78	92	91	86
ER	100	97	100	100	61	56	78	77	100	97	91	89	100	94	87	90
FM	61	69	76	76	61	72	70	67	72	78	82	84	89	92	85	88
GM	56	78	78	78	56	64	63	57	61	44	59	53	67	83	82	85
GV	50	50	48	45	33	50	56	63	61	61	57	65	83	81	80	78
IM	78	81	81	81	72	100	96	92	89	83	87	81	100	100	100	96
JB	72	86	85	82	83	86	87	86	94	83	89	85	100	92	94	91
LT	56	64	65	63	50	50	57	60	61	72	65	66	44	50	56	52
MT	67	58	65	70	72	72	81	70	72	75	70	73	67	75	80	79

**Fig 5:**
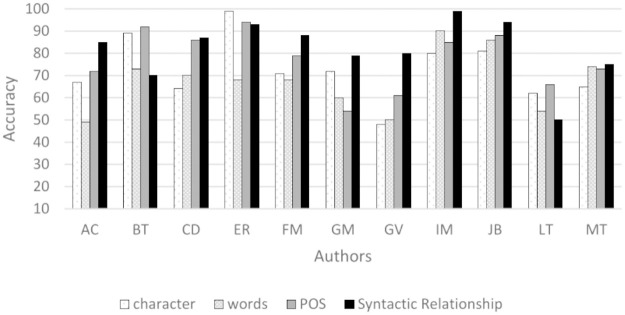
Average accuracy (%) of 3-grams using LSA features.

Fig 5 shows the average accuracy achieved for different text lengths and 3-grams. Except for BT and LT, the syntactic relationship 3-grams achieve the highest accuracy.

### Text length analysis

**Fig 6:**
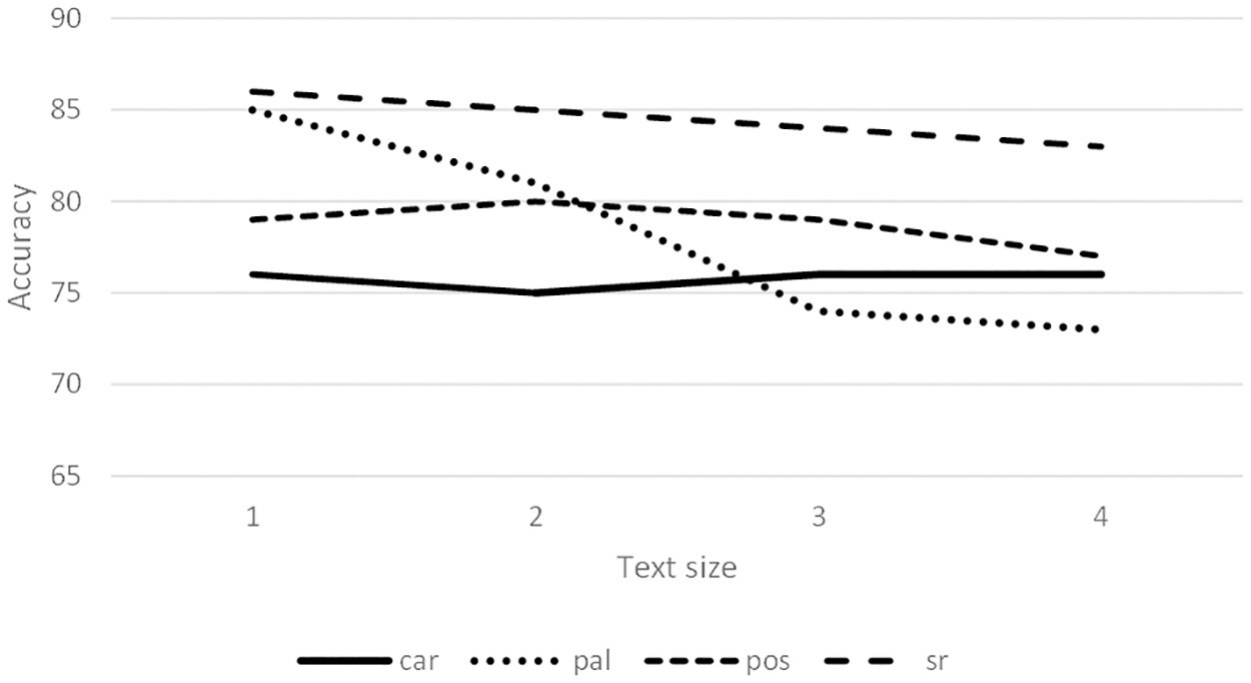
Average of the accuracy (%) using all features.

**Fig 7:**
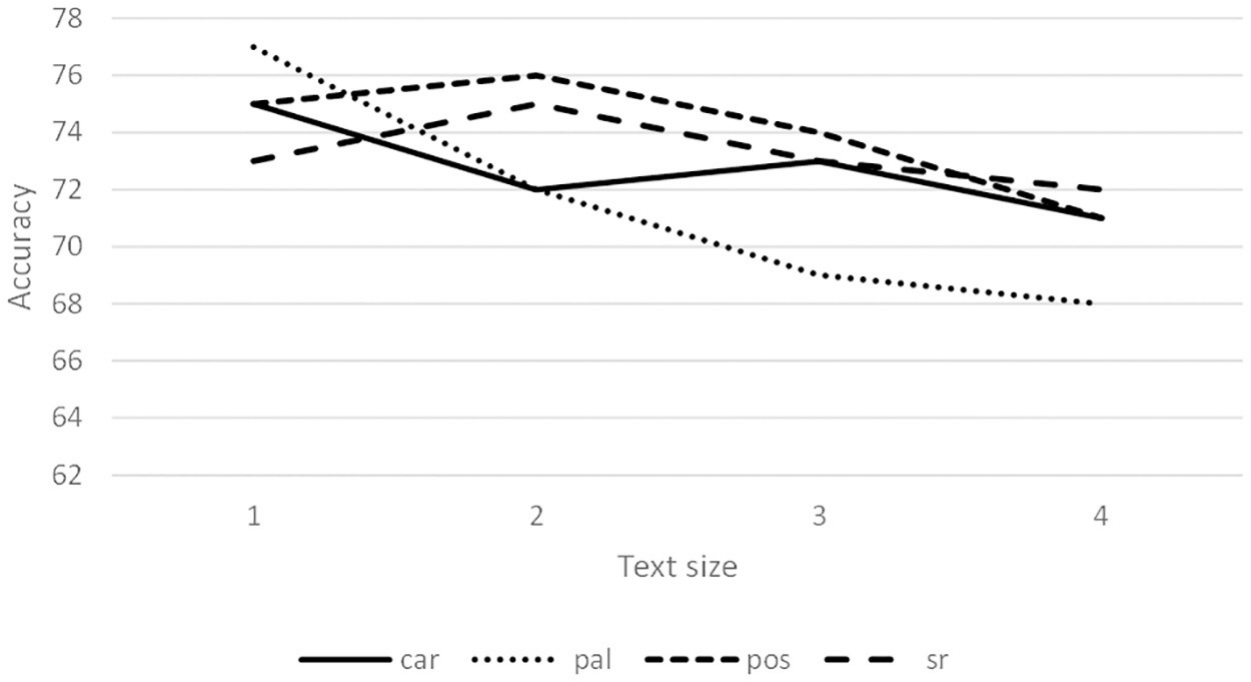
Average of the accuracy (%) using PCA features.

Fig 6 shows the accuracy of different text lengths using ALL features. Syntactic relations 3-grams achieved the best results in complete novels (1), followed by POS tag and character 3-grams. Word 3-grams obtained 85% in complete novels; this value decreased as the length of the texts also decreased. Fig 7 shows the results of different text lengths using PCA features. POS tag and syntactic relationship 3-grams showed the best results in half of the novels (2). Character and word 3-grams had the best accuracy in complete novels (1).

**Fig 8:**
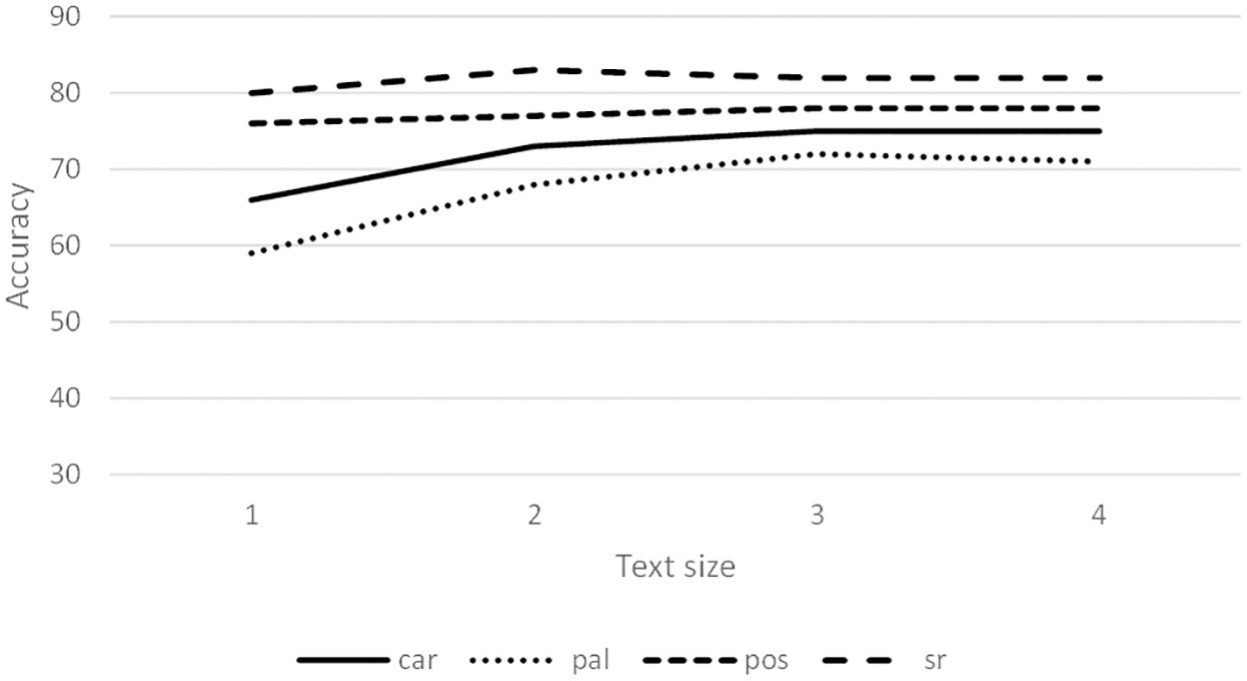
Average of the accuracy (%) using LSA features.

**Fig 9:**
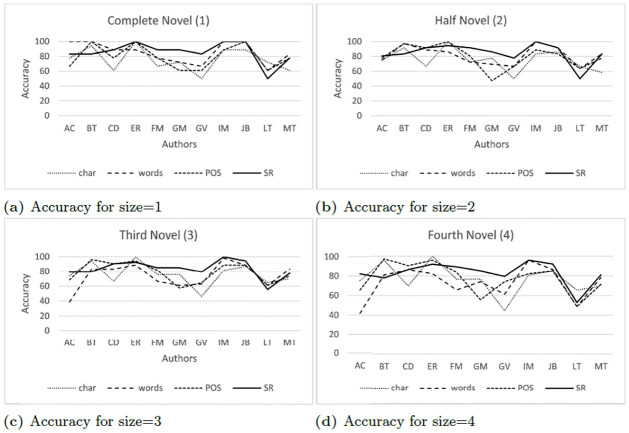
Accuracy (100%) obtained for different sizes.

Fig 8 shows the results for different text lengths using the LSA features. The best performance was syntactic relationship 3-gram with at least 80% accuracy, followed by POS tags, character and words 3-grams. Complete novels (1) had slightly lower percentages than the rest of the blocks. Fig 9 shows the accuracy obtained for different sizes and style markers. Higher scores were obtained when complete novels were used.

Table 11 shows the accuracy obtained using different 3-grams and complete novels. Syntactic relationship 3-grams improved traditional 3-grams in 50% of the authors. One interpretation of these results is that, over time, the way of structuring sentences and the frequency of use of some syntactic structures by some authors changed gradually, allowing classifiers to identify such changes. Similarly, the frequencies of use of n-grams of characters, words and POS tags were also significant for authors such as BT, ER, IM and JB achieving 100% accuracy in at least one category.

**Table 11:** Accuracy (%) using 3-grams in complete novels using ALL features.

Authors	char	words	POS	Syntactic Relationship
AC	78	100	67	83
BT	94	100	100	83
CD	61	89	78	89
ER	100	89	100	100
FM	67	78	78	89
GM	72	72	61	89
GV	50	67	61	83
IM	89	100	89	100
JB	89	100	100	100
LT	72	61	61	50
MT	61	83	78	78

To achieve a more reliable writing style change analysis, characteristics independent of the topic of the text should be used. In order to compare the performance of our syntactic feature, additional experiments were performed using words and POS n-grams with n = {1,2,3}. Table 12 shows the results obtained from these experiments using complete novels. In authors such as BT, ER and JB, the combination of words (1+2+3)-grams achieved good results, even better than the 3-gram syntax. Contrary, the authors AC, FM, GM, GV show the lowest accuracy in traditional 3-grams. However, in Syntactic Relationship 3-grams, the same authors achieve their best results. For authors IM and JB, Syntactic Relationship and word 3-grams obtain 100% accuracy. Finally, the author LT obtained only 50% accuracy in Syntactic Relationship 3-grams.

**Table 12:** Results for different types and sizes of n-grams in complete novels using ALL features.

Authors	POS n-grams			words n-grams			3-grams			
	n = 1	n = 2	n = {1,2,3}	n = 1	n = 2	n = {1,2,3}	char	words	POS	Syntactic Relationship
AC	67	78	78	67	56	56	78	100	67	83
BT	89	89	89	100	100	100	94	100	100	83
CD	61	72	72	61	83	67	61	89	78	89
ER	100	100	100	100	100	100	100	89	100	100
FM	89	78	89	56	56	56	67	78	78	89
GM	72	67	72	50	56	44	72	72	61	89
GV	67	61	67	67	56	67	50	67	61	83
IM	89	83	89	67	72	67	89	100	89	100
JB	100	100	100	100	100	100	89	100	100	100
LT	72	56	72	83	67	78	72	61	61	50
MT	67	72	72	67	83	67	61	83	78	78

### Analysis of ALL, PCA and LSA features

**Fig 10:**
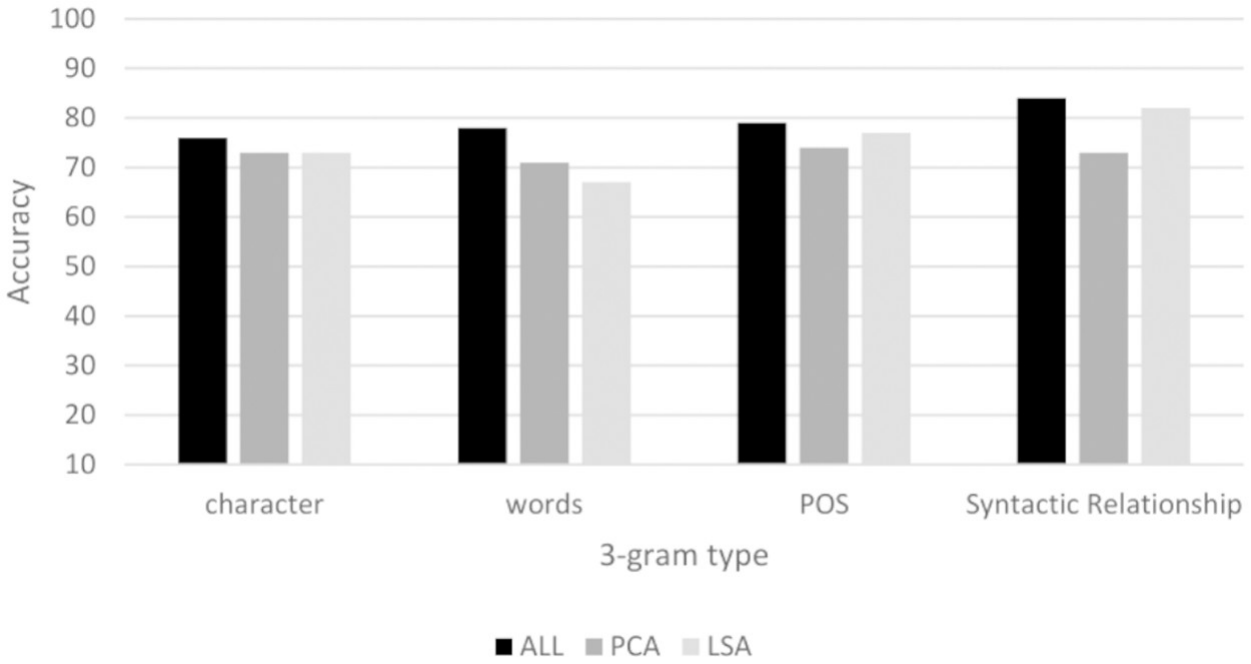
Accuracy (%) averages in models with and without a dimension reduction.

Fig 10 shows that ALL features perform better than PCA and LSA features. It seems that the dimension reduction process causes the loss of stylistic information of the authors.

### Precision, recall and F1

This section includes precision, recall and F1 averages using All features. Intuitively, precision is the ability of the classifier not to label as positive a sample that is negative and recall is the ability of the classifier to find all the positive samples https://scikit-learn.org/stable/. For both metrics, the best value is 1 and the worst value is 0. F1 score is the weighted average of Precision and Recall. Table 13 show the averages obtained in the data. Labels **p** and **r** represent precision and recall. It is difficult to observe a pattern due to the 3-gram types. However, the authors GM, LT and MT mostly show values lower than 65%. It should be noted that authors FM and LT show a substantial improvement in syntactic relationships 3-gram. The authors ER, IM, JB and LT achieve F1 greater than 90%. In general, F1 is greater than 70% in all 3-grams but it is also observed that in words 3-grams, is inferior to the other 3-grams.

**Table 13:** Precision, recall and F1 in ALL features.

Authors	char			words			POS			Syntactic relationship		
	p	r	F1	p	r	F1	p	r	F1	p	r	F1
AC	0.58	0.97	0.72	0.26	0.44	0.33	0.61	0.86	0.70	0.58	0.75	0.60
BT	0.93	1.00	0.96	0.77	0.78	0.71	0.82	0.86	0.83	0.46	0.61	0.49
CD	0.69	0.86	0.71	0.61	0.78	0.67	0.90	0.94	0.91	0.75	0.83	0.75
ER	0.86	1.00	0.91	0.55	0.67	0.55	0.80	1.00	0.88	0.96	1.00	0.97
FM	0.64	0.78	0.69	0.55	0.75	0.61	0.88	0.86	0.82	0.83	1.00	0.88
GM	0.63	0.72	0.65	0.44	0.56	0.47	0.42	0.53	0.44	0.67	0.58	0.60
GV	0.46	0.89	0.61	0.81	0.94	0.84	0.86	0.97	0.91	0.61	0.78	0.66
IM	0.84	0.94	0.88	0.59	0.67	0.61	0.71	0.75	0.70	0.96	1.00	0.97
JB	0.78	1.00	0.86	0.93	1.00	0.96	0.62	0.75	0.66	0.87	1.00	0.92
LT	0.67	0.67	0.66	0.59	0.39	0.43	0.72	0.42	0.50	1.00	0.89	0.92
MT	0.73	0.50	0.57	0.33	0.36	0.34	0.77	0.56	0.58	0.73	0.69	0.67

### PCA and ROC curve visualization

**Fig 11:**
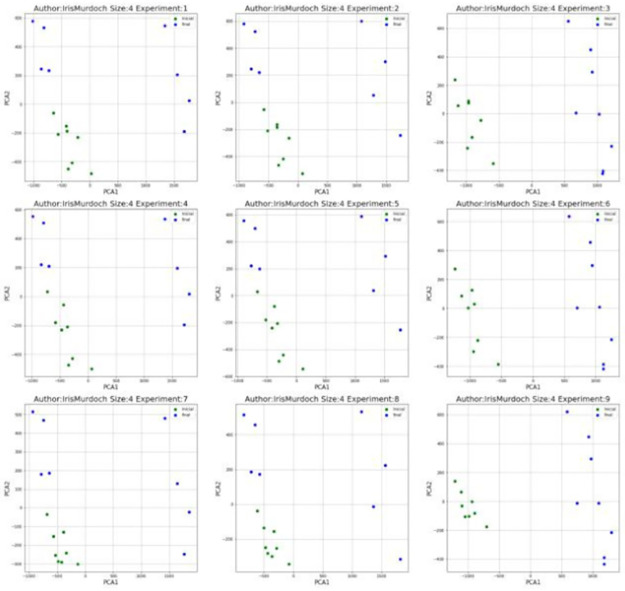
PCA visualization of Iris Murdoch (IM) using syntactic relationship 3-gram.

PCA transforms a high-dimensional data to low-dimensional data. This technique was applied in ALL features matrices, which contain at least 1000 features (see Table 4). The number of components used for the PCA algorithm is *k* = 2. For each author, 9 experiments were performed on each type of 3-gram. The Fig 11 shows the 2-dimensional projection of Iris Murdoch (IM) after applying the PCA algorithm using syntactic relationships and 4 blocks of text per novel. The green and blue dots are novels that belong to the *initial* and *final* stages respectively. The novels of the initial stage are grouped in the lower left area of the images, novels of the final stage are scattered in the remaining area. There is a clear separation between the two stages. The visualization shows that for this author, the metrics will show good results in the classification tests.

**Fig 12:**
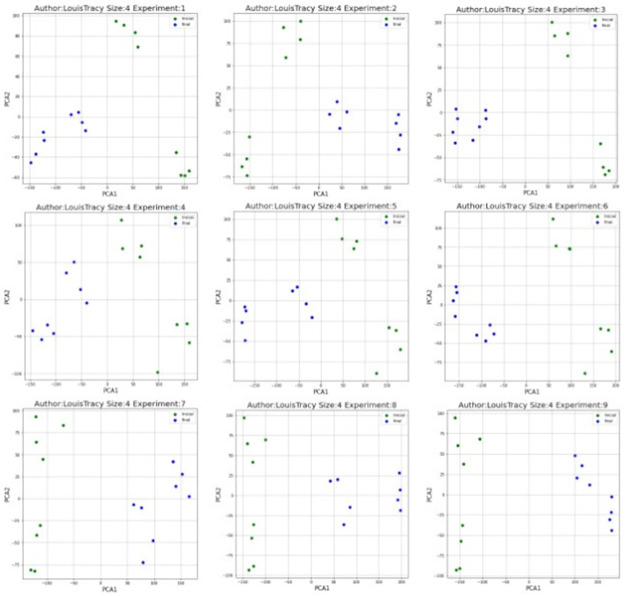
PCA visualization of Louis Tracy (LT) using syntactic relationship 3-gram.

Fig 12 shows the 2-dimensional projection of Louis Tracy (LT), both classes do not form well-defined clusters. With respect to the other authors, LT is the author who showed the lowest results in the different types of 3-grams.

**Fig 13:**
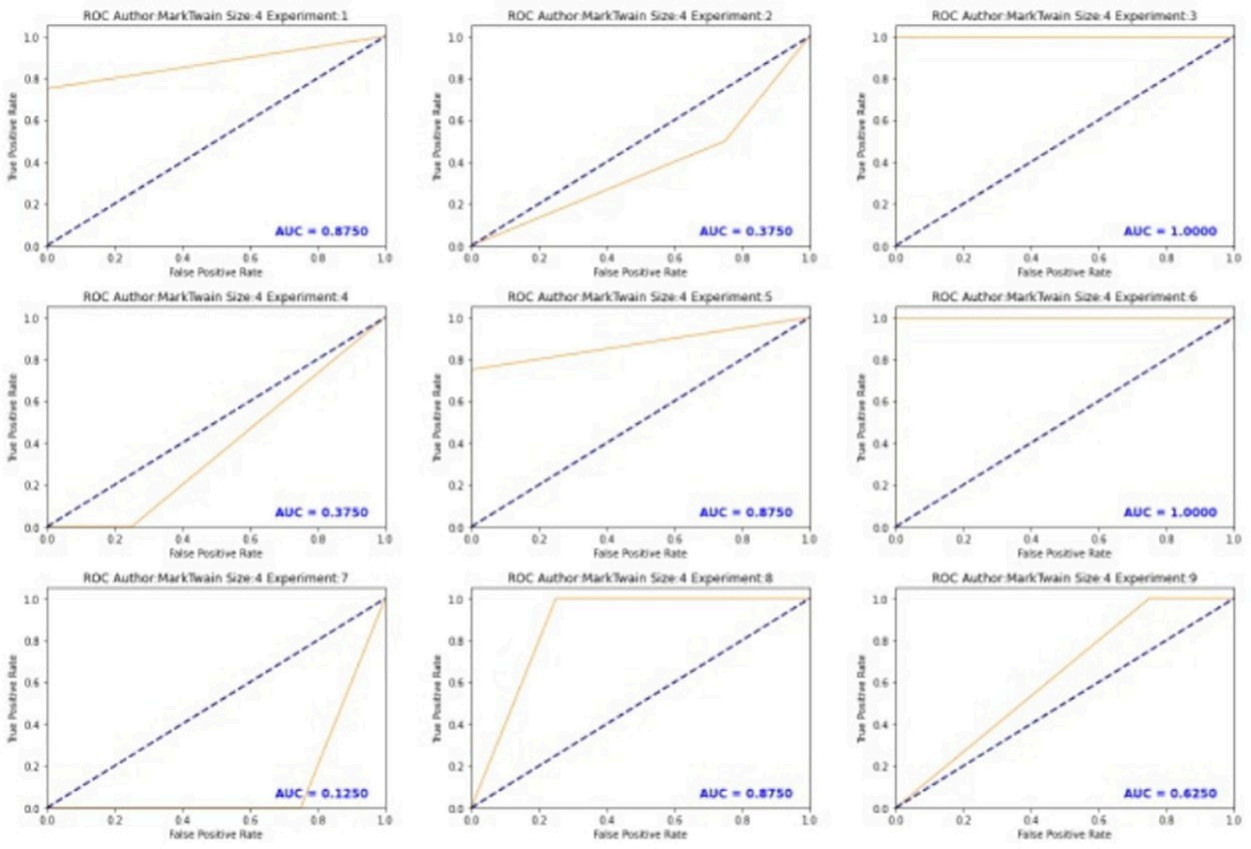
ROC of Mark Twain (MT) using syntactic relationship 3-gram.

**Fig 14:**
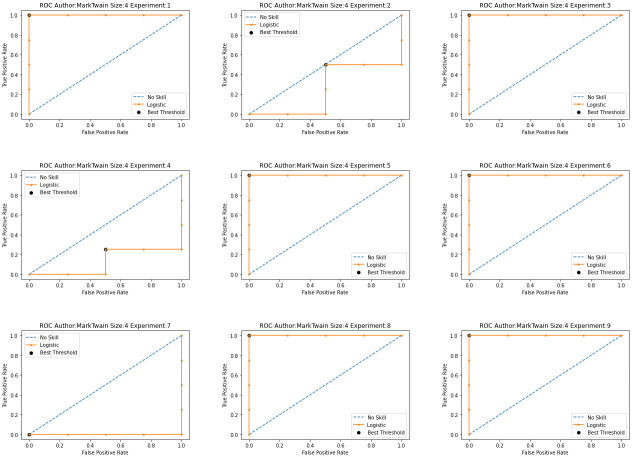
AUC of Mark Twain (MT) using syntactic relationship 3-gram via different threshold.

According to [53], performance measures such as accuracy, precision, sensitivity, and specificity depend on the discrimination threshold used to dichotomize the predicted binary outcomes. On the other hand, the area under the ROC curve (AUC) does not depend on a chosen decision threshold [54]. A random classifier is expected to give points lying along the diagonal in the ROC. If the AUC equals to 1, the classifier is expected to have perfect performance. Fig 13 shows the ROC curve and AUC for MT author and Fig 14 shows the AUC varying the threshold value. The experiments 1, 3, 5, 6, 8 and 9 show to have higher values in the AUC than the rest of configurations.

### Statistical analysis

The hypothesis test was performed using One-sample T-Test. This test was used to determine if the LR classifier predictions were statistically significant relative to a hypothetical 50% mean. Thus, the null hypothesis was *H*0 : $\bar{x}=50$ and the alternate hypothesis as *H*1 : $\bar{x}>50$. According to Table 6, for each author, nine predictions were made. Since this is one sample T-Test, the Degree of Freedom (DOF) is 8. The value of alpha was (*α* = 0.05) to meet 95% confidence level.

Using the DOF, alpha and confidence level were found in the T-Table that T-Critical was equal to 1.860. We used the *ttest*_1*samp*() function from the *scipy.stats* library to conduct this T-Test. This function returns the parameters *tscore* and *pvalue*. In every experiment, the values of these parameters change because they depend on the sample mean and sample standard deviation of each particular case.

If the *pvalue* is less than *α* then the null hypothesis is rejected, otherwise fail to reject the null hypothesis. Reject the null hypothesis means that results are statistically significant then there is enough evidence to conclude that the accuracy of the classifier is greater than 50%.

Table 14 shows the results of the hypothesis test. The value 1 indicates the cases in which the null hypothesis is rejected, i.e., H1 is accepted. It is observed that for the GM, GV, LT and MT authors the mean accuracy is not statistically significant. In such cases, there is not enough evidence to reject H0 (fail to reject). For some authors the change in writing style based on the 3-gram frequency is more evident than in others.

**Table 14:** One-sample T-test results for different types of 3-grams.

Author	char				words				POS				SR			
	1	2	3	4	1	2	3	4	1	2	3	4	1	2	3	4
AC	1	1	1	1	0	0	0	0	1	1	0	0	1	1	1	0
BT	1	1	1	1	1	1	1	1	1	1	1	1	1	0	0	0
CD	0	1	1	1	1	1	1	1	1	1	1	1	1	1	1	1
ER	1	1	1	1	1	1	1	0	1	1	1	1	1	1	1	1
FM	0	1	1	1	1	1	1	1	1	1	1	1	1	1	1	1
GM	1	1	1	1	0	0	0	1	0	0	0	0	1	1	1	1
GV	0	0	0	0	1	1	1	1	1	0	0	1	1	1	1	1
IM	1	1	1	1	0	1	0	0	1	0	1	0	1	1	1	1
JB	1	1	1	1	1	1	1	1	1	1	1	1	1	1	1	1
LT	0	0	0	0	1	0	1	1	1	1	1	1	0	1	1	1
MT	0	0	1	1	0	0	0	0	1	1	1	1	1	1	0	0

## Discussion

This article discusses the task of detecting changes in writing style over time and evaluates the efficiency of syntactic style markers. In a previous study [39], it was shown that syntactic n-grams obtain competitive results with respect to traditional n-grams. Unlike the previous work, only the initial and final stages were used, and the intermediate stage was removed. Since the time difference in the publication of novels is important, it was decided to use only the initial and final stages. With this modification, it was expected that the change in writing style would be more noticeable for all authors (the time gap between both stages was at least 5 years). By removing the middle stage, we achieved higher overall performance metrics for authors compared to previously published studies [39], regardless of whether complete or half novels were used.

In addition, the number of authors in the corpus was increased (4 more authors), the usefulness of a second feature reduction method (LSA) was evaluated, and smaller sizes for the instances (third and fourth novels) were tested. Four types of 3-grams were used that covered different aspects of the language: characters, words, POS tags and syntactic n-grams.

In this study, GV and LT showed averages of accuracy just above 60% in the different types of 3-grams (see Tables 8–12). The rest of the authors reported averages of accuracy greater than 80%. The performance of the syntactic 3-grams in ER, IM and JB is superior to the other 3-grams, they show 100% accuracy in some of the configurations.

Experiments also showed that blocks of complete novels lead to results that are slightly superior than smaller blocks (see Figs 6–8). Dividing the novels into smaller samples allows an increase in the number of instances for training and testing the learning algorithms. However, the amount of text in each sample decreases for each instance.

We evaluated the convenience of using dimension reduction techniques (PCA and LSA algorithms) for this task. These algorithms reduce a large number of features to a minimum set and it is expected that when applied, the performance metrics will improve substantially. Table 4 shows that in 3-grams of words, all authors had at least 1,000 features. In the other 3-grams, they had at least 3,000. Most of these features had a low frequency (frequency = 3). Fig 10 shows that the results of the models created with the PCA and LSA algorithms were not superior to the models without reduction of dimensions. There is no rule to determine what is the appropriate number of dimensions (*k* value). In this experiment, PCA results with *k* = 2 and LSA with *k* = {4, 8, 12, 16} showed to be better than other configurations. For LSA, this values represents the number of examples in training set.

The results obtained with syntactic relationship 3-grams show that they are a viable option for detecting writing style changes over time, since their performance was the same and in many cases, better than the other proposed n-grams. In addition, they are robust to changes in the document topics. Syntactic n-grams can also be composed of words and POS tags. These factors allow them to identify usage patterns that are not visible at the surface level of the text. Nevertheless, we leave the analysis of the performance of these types of syntactic n-grams for future work.

## Conclusions

The proposed method proved that it is possible to detect changes in writing style over time by means of the frequency of use of n-grams and machine learning strategies. The conducted experiments revealed that the classifiers can learn the style of the authors for the proposed time stages, which indicates changes in the styles of the authors. However, they depended on the author. In some of them, the change is noticeable but in others it is not.

Through the proposed n-grams, patterns can be detected at different language levels. In the English language, character 3-grams can detect certain sequences, for example, *ing* related to gerund verbs and contractions like *don’t* or *can’t*. The word n-grams can detect sequences like *as well as* or *as known as* (collocations). The POS tag n-grams show the grammatical category of each word, a sequence like *DT* + *JJ* + *NN* indicates that the sentence contains an article, followed by a qualifying adjective and a noun. Syntactic n-grams are obtained by traversing the dependency tree of a sentence. Therefore, sequences that are not linear can be identified. This characteristic that makes them an ideal candidate for a reliable writing style analysis.

The representation of the author’s style based on vocabulary is useful for the task. However, it has the disadvantage that the vocabulary used by the author changes depending on the theme of the novel. The changes identified by means of words should not be interpreted as a change of style.

Stylometric features based on syntactic information showed results similar or even better to the n-grams of characters, words and POS tags. These markers reveal very different patterns than those that occur when text is parsed in its linear form. The syntactic information of a sentence is shown in the form of trees that show that even words distant from each other are related by some dependency relations. This fact allows discovering new patterns in writing style. It should be clarified that the change in writing style that is based on the frequency of use of the n-grams. The fact that the syntactic 3-grams show low accuracy means that at the syntactic level there is not enough information for the classifier to differentiate between the initial and final stages.

Dimension reduction techniques should be applied with caution in our task since an improvement in the performance is obtained very rarely. Based on these experiments, we can conclude that for classification tests, the use of these reduction algorithms is not recommended.

Experiments varying the number of sentences of input text to the classifier were performed in order to determine how the amount of data affect the performance of the proposed method. The case with the highest quantity corresponds to 1,000 sentences, while the case with the least quantity corresponds to 500 sentences. If an author has a considerable number of novels, evaluating them without dividing them is the best option. Otherwise, division into proportional parts is suggested. The minimum amount of text (per number of sentences) will depend on the writing style of each author.

The frequency of use of n-grams over the time and supervised machine learning algorithm showed that these approaches are applicable to the problem of detection of change of style, obtaining competitive results (in general, an efficiency higher than 70%). The experiments carried out showed that it is possible to detect changes in an author’s style over time due the frequency of use of n-grams.

## Supporting information

S1 File Dataset of novels of 11 native English-speaking authors. (ZIP)
